# Singing-Related Activity in Anterior Forebrain of Male Zebra Finches Reflects Courtship Motivation for Target Females

**DOI:** 10.1371/journal.pone.0081725

**Published:** 2013-11-29

**Authors:** Mai Iwasaki, Thomas M. Poulsen, Kotaro Oka, Neal A. Hessler

**Affiliations:** 1 Brain Science Institute, RIKEN, Wako-shi, Japan; 2 Biosciences and Informatics, Keio University, Yokohama, Japan; Claremont Colleges, United States of America

## Abstract

A critical function of singing by male songbirds is to attract a female mate. Previous studies have suggested that the anterior forebrain system is involved in this courtship behavior. Neural activity in this system, including the striatal Area X, is strikingly dependent on the function of male singing. When males sing to attract a female bird rather than while alone, less variable neural activity results in less variable song spectral features, which may be attractive to the female. These characteristics of neural activity and singing thus may reflect a male's motivation for courtship. Here, we compared the variability of neural activity and song features between courtship singing directed to a female with whom a male had previously formed a pair-bond or to other females. Surprisingly, across all units, there was no clear tendency for a difference in variability of neural activity or song features between courtship of paired females, nonpaired females, or dummy females. However, across the population of recordings, there was a significant relationship between the relative variability of syllable frequency and neural activity: when syllable frequency was less variable to paired than nonpaired females, neural activity was also less variable (and vice-versa). These results show that the lower variability of neural activity and syllable frequency during directed singing is not a binary distinction from undirected singing, but can vary in intensity, possibly related to the relative preference of a male for his singing target.

## Introduction

For both males and females, interaction with individuals of the opposite sex is critical for successful reproduction. In addition, a subset of species further expands such interaction to prolonged pair bonding [1],[2]. Such social monogamy is especially widespread among songbirds (∼90% of species [3]). Within monogamous pairs, though, individuals occasionally choose to engage in extra-pair interactions [4]. Such decisions, which can influence an individual's reproductive success and maintenance of their pair-bond, are among the most important that females and males make.

In zebra finches, males court females by producing a stereotyped "directed" song, in response to which the female can choose to either continue the courtship interaction, or reject the male. In non-courtship contexts, males also produce a similar "undirected" song, apparently without a specific target [5],[6]. While both song types are very similar acoustically, directed songs can be distinguished by a lower level of variability of fine frequency control, and a slightly higher singing tempo [7],[8]. Importantly, female zebra finches can discriminate between these songs, and prefer to approach directed ones [9].

Production of both directed and undirected songs is controlled by a specialized brain network known as the song system that is only present in songbirds [10]. Activity in one subset of the song system, the anterior forebrain pathway (AFP), determines whether singing output is directed or undirected. The AFP network consists of the basal ganglia Area X, its efferent dorsal lateral nucleus of the medial thalamus (DLM), and the lateral magnocellular nucleus of the nidopallium (LMAN), which provides input to the singing premotor robust nucleus of the arcopallium (RA). This input controls a characteristic feature of directed singing, reduced variability of syllable fine frequency control. During undirected singing, LMAN projection neurons fire with a relatively variable pattern related to song elements, while during directed singing this neural activity is much more stereotyped. Critically, inactivation or lesions of LMAN reduce the variability of all singing, causing undirected songs to become similar to directed ones [11],[12].

This control of neural variability in AFP nuclei and of courtship-related features of singing appears regulated by dopaminergic input from the ventral tegmental area [13],[14],[15],[16],[17]. Stronger activation of dopaminergic neurons during directed singing causes higher levels of dopamine release in AFP nuclei, and reduces the level of neural variability during courtship. Thus, in contrast to undirected singing, female-directed courtship singing is associated with strong activation of dopaminergic systems that are related in mammals with motivation and goal-directed behaviors [18],[19]. When males sing during courtship of females, but not when they sing while alone, the level of neural activity [14] and of activity-dependent gene expression [15] in VTA is selectively modulated, and higher levels of dopamine are present in Area X [13]. Further, block of dopamine receptors restricted to Area X causes directed singing to become more similar in variability to undirected singing [17].

The modulation of neural activity and song features present in directed singing, and absent in undirected singing, reflects in some way the male's female-directed courtship behavior. Here, we tested whether AFP activity and singing features were consistently less variable, characteristic of courtship singing, when males sang to females of a particular pairing status. For each male, a female was operationally defined as "paired" or "nonpaired" based on whether or not they had previously cohabitated with him for 1-2 weeks. We found that across all recordings, variability of both AFP activity and songs were not consistently dependent on female pairing status. However, within individual recordings, the variability of neural activity was closely related to syllable variability. These results highlight the complexity of courtship interactions for both males and females.

## Materials and Methods

### Ethics Statement

All procedures were reviewed and approved by the RIKEN Animal Experiments Committee (Approval ID: H23-1-223).

### Pairing procedure

Eight adult zebra finches (over 120 days old, 4 each male and female) were used in these experiments. For each opposite sex pair, birds were taken from a group housing cage in an open room containing other zebra finches, and moved into a cage (27 x 27 x 20 cm) in a sound attenuation chamber (60 x 50 x 50 cm). Males were moved into the cage one day before females. Following 8-13 days of housing together, all pairs had attained some level of pair-bonding, as defined by the occurrence of clumping behavior and allopreening [5],[20],[21],[22],[23]. Both birds were then transferred into separate small cages (20 x 20 x 20 cm) in sound attenuation chambers containing no other birds.

### Surgical procedure

1-4 days after separation from the female, males were implanted with a miniature movable microdrive, as described previously [24]. In brief, under inhalation anesthesia (isoflurane), a movable microdrive containing two insulated tungsten electrodes (∼3Mohm, Microprobe) was positioned with electrode tips 1.3 mm below the surface of the brain, and fixed to the skull with 5-minute epoxy (Devcon).

### Recording procedure

**Figure 1:**
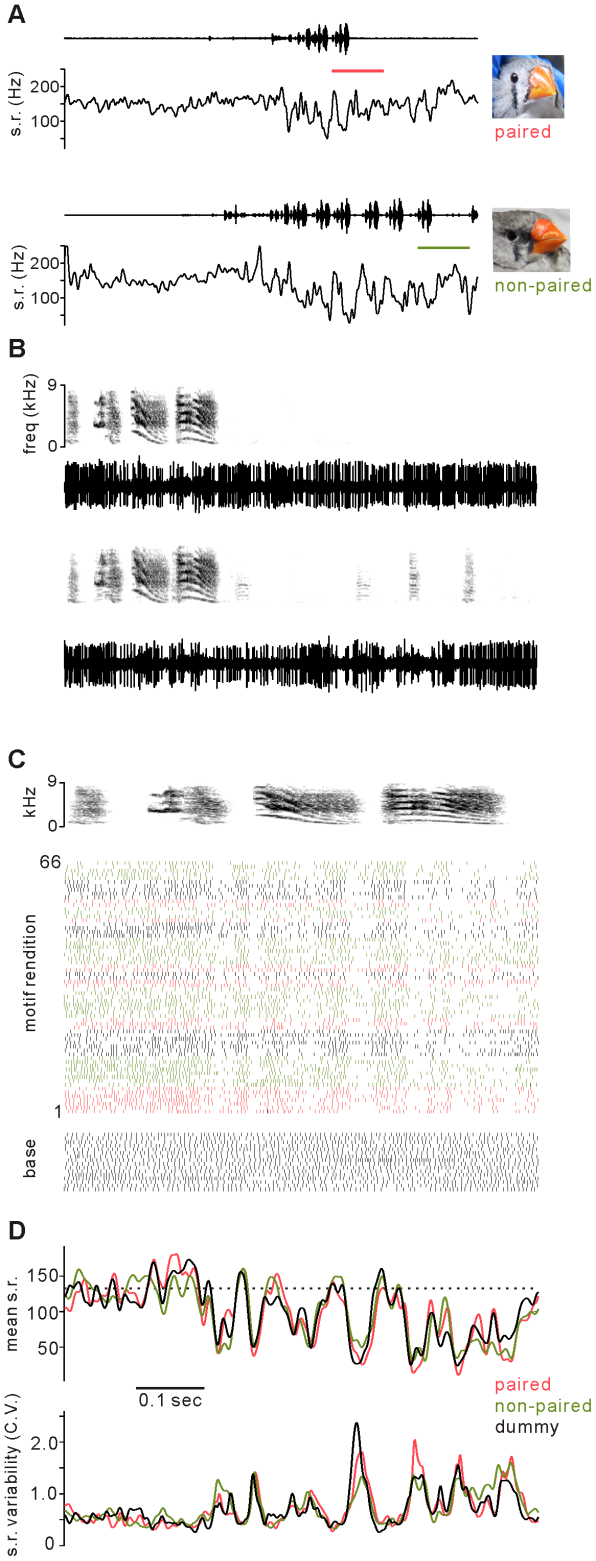
Example unit activity before and during singing directed to paired, nonpaired, and dummy females. A, Concurrently recorded sound oscillogram (upper traces) and instantaneous firing rate of representative Area X unit taken from epochs including non-singing and singing to either a paired female (upper panels) or a nonpaired female (lower panels). **B**, Expanded views of sound (upper panels, spectrogram) and neural activity (lower panels, activity waveform) in periods of panel **A** indicated by orange (paired) and green (nonpaired) color bars, which contain similar singing and non-singing periods for both females. **C**, Activity of unit during multiple renditions of the characteristic song motif directed to different female classes. Upper spectrogram is a representative song motif. For each successive motif rendition, unit firing time is indicated by vertical tick. Raster of activity during equal duration epochs when male did not sing is indicated by base. **D**, Average firing rate for each msec of analysis epoch (upper) and variability of firing rate across all renditions to each female class (C.V.  =  S.D./mean).

After 2-3 days of recovery, a flexible wire lead containing a headstage (Plexon, Dallas TX, USA) was connected to the microdrive, and signals from the electrode were passed through a rotating commutator (Dragonfly, Ridgeley WV, USA), amplified, and digitized at 40 kHz, along with the acoustic signal in the chamber (Plexon). During each recording session, electrodes were slowly lowered until apparently isolated single-unit activity was present. At intervals of about ten minutes, the door of the sound attenuation box was opened and a small cage containing either the female who had been paired with the male, a female who had not been paired with the male, or a painted dummy model of a female zebra finch or java sparrow was placed next to the male's cage. For most recording sessions, either 2 or 3 nonpaired females were used, including females of similar and different color morphs (e.g., Fig. 1A,B), but in 4 sessions only a single nonpaired female was used (3 similar color, 1 different). Females that were operationally defined as "nonpaired" for an individual male were used as "paired" females for other experimental males, and thus had recent (within 3 weeks) experience of living with a male, and of being used as a singing stimulus. Subsequent behavior of both birds was monitored by video camera, and often included directed singing of the male towards the female. The caged female was removed after about 2-3 minutes, whether the male sang or not. Recording sessions occurred over a period of 2-9 days, from 3-14 days after males were separated from paired females. Behavioral data were analyzed only from recording sessions in which neural activity was recorded.

### Analysis

After recordings were completed, unit activity was sorted with Offline Sorter (Plexon). Unit isolation was confirmed as > 99% of ISI's were greater than 1 msec [25]. All subsequent analysis was performed with Matlab (Mathworks, Natick, MA, USA).

For each recording session, productions of a typical song motif (containing 4-6 syllables, of duration 475 - 769 msec) were detected by matching a template of sound amplitude level to the acoustic signal recorded along with neural recordings. In order to account for some variability in motif duration, for each song motif the estimated deviation of motif initiation and termination time from the average motif initiation and termination time was determined by visual comparison of the smoothed rectified waveforms of both. Following this, unit firing times for each motif were shifted to account for these deviations. In brief, spikes occurring at motif initiation were shifted forward or backward in time according to the estimated deviance of a motif's onset from the average motif, and spikes occurring at the motif termination were shifted according to the estimated deviance of the motif termination time. Spikes within motifs were shifted proportional to their position relative to motif initiation and termination. After shifting, the instantaneous spike rate for each song motif rendition was estimated by smoothing with a 10 msec Gaussian window. While this method assumes that motif temporal variability is uniform across all song elements, it could fail to fully characterize variability in timing of individual syllables in motifs. However, frequent vocalizations of females made accurate identification of all syllable boundaries difficult.

Two measures of variability of unit activity with repeated song motifs were calculated for songs produced to each female class [24]. The cross-rendition coefficient of variance (C.V.) was calculated by dividing the standard deviation of the mean instantaneous spike rate by the mean spike rate, in 1 msec intervals. For each recording session, the mean C.V. of all song motifs was used for comparisons between female classes.

To quantify within-rendition variability, the cross-correlation (xcorr) between the instantaneous spike rate of each motif rendition and the average spike rate across all motifs was calculated.

As no clear differences were apparent in neural activity to conspecific or heterospecific model dummies (n = 3: zebra finch/java sparrow ratio of mean C.V.  =  1.03, mean xcorr  =  1.04, both ns sign rank test), results from both were combined in analyses.

Variability in production of song motif syllables for each female class was determined by measuring either the fundamental frequency of a relatively constant frequency harmonic stack, or the frequency of a tonal element [8]. For harmonic stacks, the fundamental frequency was estimated by finding the frequency of the first peak of the autocorrelation of the spectral density of a 10 msec window within the stack. For tonal elements, the frequency of the first peak in the autocorrelation of a 10 msec window was used.

Global features of singing related to timing of motif production were determined by first calculating the latency of each motif onset relative to the time at which the female cage was placed into the recording chamber. For each female presentation, the latency of the first motif produced, the number of motifs produced, the tempo of motif renditions (motif onset latency_x+1_-motif onset latency_x_) and the acceleration of motif renditions (tempo _x+1_-tempo_x_) were calculated. Examination of latencies of the first motif produced for all recordings revealed that only 3/113 were above 40 seconds, and all others below 20 seconds. Thus, only those less than 20 seconds were used for calculating mean initial latency values. Quantification of the remaining features was restricted to singing bouts beginning prior to 20 seconds, as well.

In order to focus on motifs produced within the initial continuous singing bout, we further examined the distribution of singing tempos. The majority of successive motifs were produced with intervals of 4.2 seconds or less, with progressively fewer of longer intervals (11/468 > 4.7 sec, 12 > 4.2 sec, 14 > 4 sec, 18 > 3.8 sec). Therefore, we limited quantification of motif number, singing tempo, and acceleration to motifs produced prior to the first motif interval greater than 4.2 seconds. Within this subset of acceleration values, the mean acceleration variability was quantified by calculating by the S.D. of acceleration values, and the mean level of acceleration alternation was quantified by the percentage of successive acceleration values that were of opposite sign (percentage of successive acceleration values that crossed the zero level).

For quantification of average motif duration, initiation and termination times were estimated by visual comparison of each rendition's sound amplitude waveform to the average motif rendition. Variability of motif durations calculated in this way were in the range of those reported in previous studies (motif duration C.V.  =  0.007 - 0.013).

Because undirected singing was produced in only 3 recording sessions, and consisted of few motif renditions (5-9), we did not further analyze this in detail.

### Statistical analysis

The majority of analyses tested whether characteristics of neural activity and singing features were different during singing directed to paired females than other female types. Thus, we performed statistical tests comparing neural activity and singing between paired females and nonpaired females, and between paired females and dummy females. Because data from paired females were used for 2 comparisons, we used a conservative significance level of p  =  0.01 for all tests.

**Figure 2:**
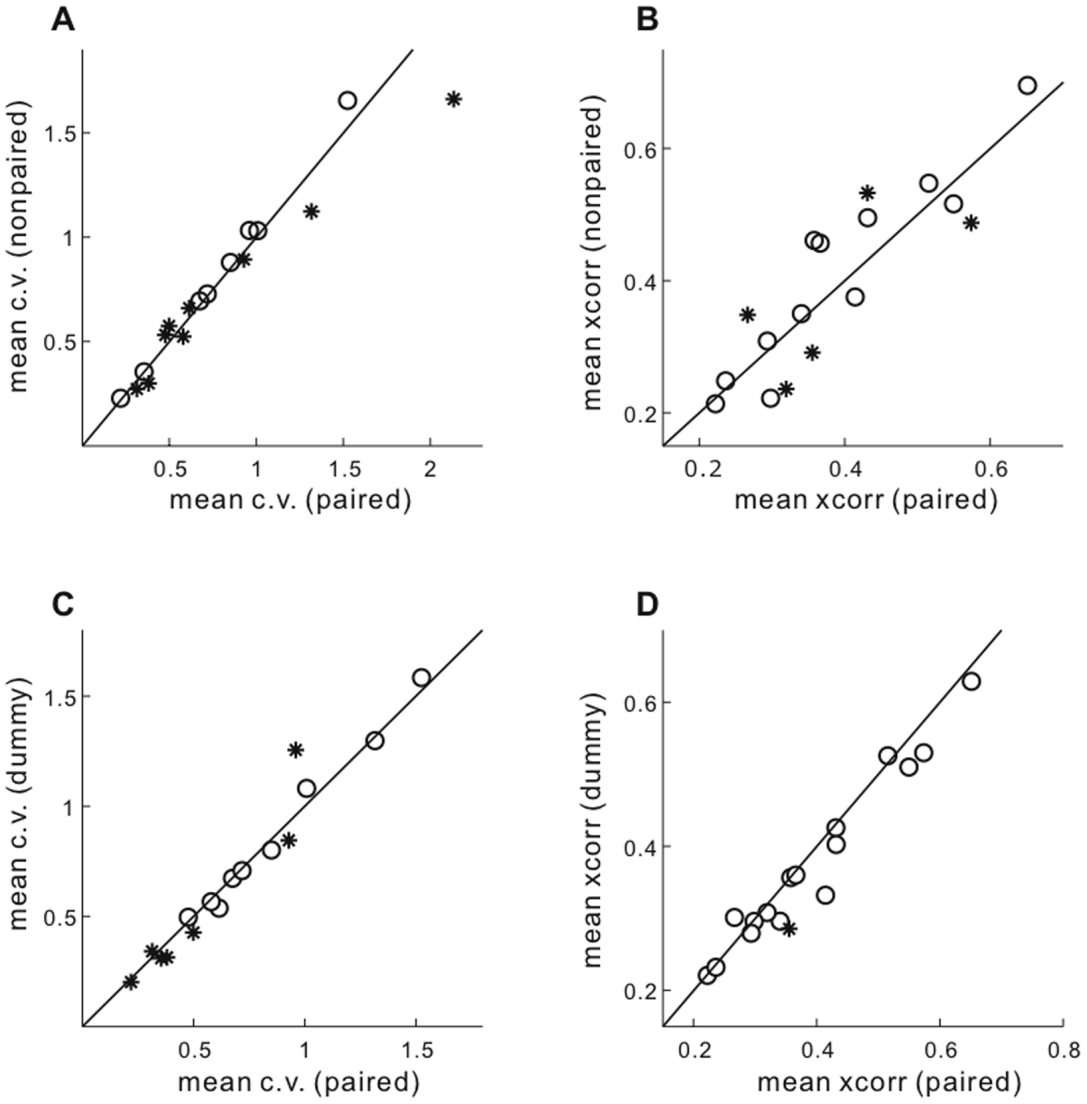
Variability of unit activity during song motifs directed to different female classes. A - B, Comparison between paired and nonpaired females. Mean cross-rendition variability (C.V., left panel) and mean within-rendition variability (xcorr, right panel). Across the population of recordings, variability during motifs directed to paired and nonpaired females was similar. Individual recording sessions in which mean C.V. or mean xcorr were significantly different indicated by *. **C - D**, Comparison between paired and dummy females, as in panels **A - B**.

For tests comparing neural results between paired vs. other female classes, we used the average values of cross-rendition C.V. and within-rendition xcorr for each recorded unit. A test of whether neural variability of the population of units was different between paired and other females was made with a paired statistical test (e.g., Fig. 2A).

**Figure 3:**
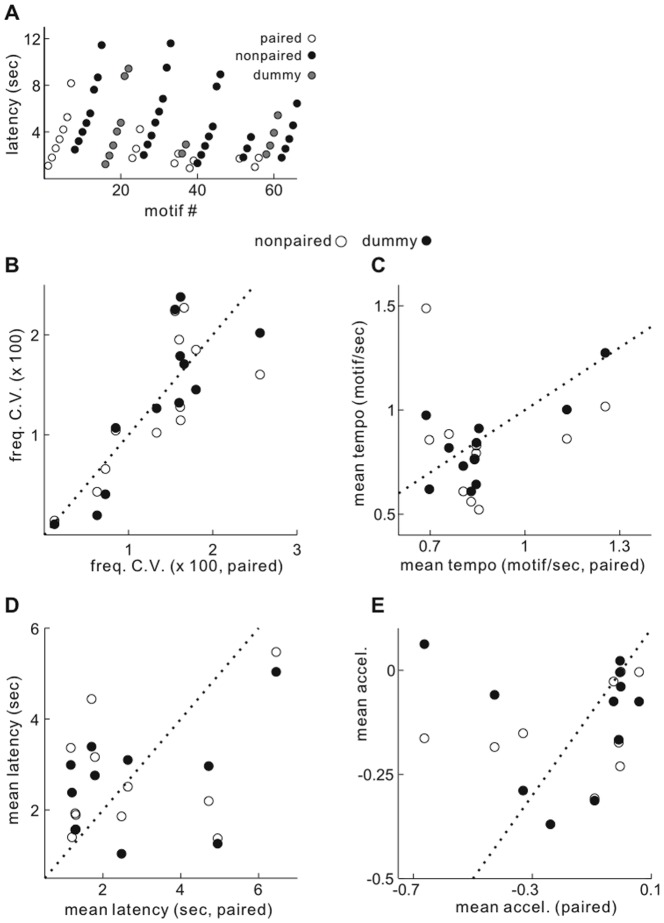
Spectral and global features of singing directed to different female classes. A, Timing of song motif production in a representative recording session used for example Figure 2. Latency of each motif initiation during song bouts directed to paired (open circles), nonpaired (black circles), and dummy females (gray circles). **B-E**, Mean of song features within each recording session for paired female plotted versus both nonpaired (open circles) and dummy (black) females. For each panel, one point summarizes results from singing of one bird in one recording day. **B**, Mean variability of syllable fundamental frequency. **C**, Mean tempo of motif production over all singing bouts. **D**, Mean latency of first motif produced. **E**, Mean acceleration of motif production.

For tests comparing singing features, we used the average values within a single day of recording for each bird, because multiple units were recorded on some days. Tests comparing singing features between paired and other females were made using paired statistics, as above (e.g., Fig. 3C).

The choice of parametric or non-parametric tests for each comparison was based on a Lilliefors test of normality. Those distributions consistent with normality, paired t-tests were used, and for others sign-rank tests were used.

In addition to these paired population tests comparing paired with other females, we compared measures of neural variability for each single unit, during singing to paired and other females (indicated by asterisks in Fig. 2). The comparisons of cross-rendition C.V. and within-rendition xcorr were made in different ways. Because calculation of the average cross-rendition C.V. results in a single time-series for each female type (e.g., Fig. 1C), for statistical comparison the average C.V. waveform was divided into 20 equal duration epochs (e.g., for a 1000 msec motif, 20 epochs of 50 msec duration). The average cross-rendition C.V. in each of these epochs was then used to make paired comparisons between female types, with choice of tests based on distribution normality as above. In contrast, a single value of within-rendition xcorr is calculated for each song motif. Comparisons between female classes of the resulting independent samples of within-rendition xcorr were made with unpaired statistical tests within single days. Tests between groups with normal distributions were made with t-tests, and others were made with Mann-Whitney U tests.

Unless otherwise noted, S.D. is used to indicate error values.

## Results

### A. Neural characteristics of directed singing compared between paired and other female classes

An increased variability of AFP nuclei neural activity, which is reflected in song feature variability, occurs when a male sings alone compared to when singing to a female bird [8],[26],[27]. Such reduction of neural and singing variability during directed singing is thus characteristic of the behavioral state of motivated courtship. Here, we tested whether such neural and song variability related to courtship is lower when males sing to females with whom they had previously been paired, compared to when males sing to females with whom they had not (classified as "paired" and "nonpaired" females, respectively).

Several days after males had been paired with a female zebra finch (for 1-2 weeks), single unit activity was recorded from the song system AFP nuclei Area X or LMAN (13/17 Area X). As variability of activity in both nuclei is lower during directed compared to undirected singing [26], results were combined in analyses except as noted. During each recording, cages containing the female with whom the male had been paired, a female with whom they had not been paired, or a dummy model of a female finch were placed into the recording chamber at intervals of several minutes. Males quickly oriented toward and began directing song to all female types during most such presentations (singing% of all presentations, paired  =  93, nonpaired  =  86, dummy  =  95).

Of the 13 units recorded from Area X, most were of the putative pallidal type, as indicated by their relatively high firing rate (> 58 Hz) and regular firing (median ISI C.V.  =  0.42; n  =  11) during non-singing periods, while few were of the putative striatal type (n  =  2; mean s.r. < 30 Hz, mean ISI C.V  =  1.02, [28]). Consistent with this distinction, across all Area X units there was a strong negative correlation between firing rate and ISI C.V. during baseline non-singing periods (Fig. S1A, r  =  0.81, p < 0.001, Pearson's correlation). The average firing rate of putative pallidal units was not consistently modulated during singing: both increases and decreases of activity were observed (Fig. S1B; mean singing/non-singing spike rate  =  1.08, n  =  4 < 1, n  =  7 > 1[26]). Instead, a more characteristic feature of pallidal unit activity during singing was an increase of phasic modulation from non-singing periods, including both decreases and increases of firing rate in single song renditions (resulting in a higher ISI C.V. during singing). Across all Area X units, the level of such phasic modulation was strongly correlated with the non-singing spike rate (Fig. S1C), with modulation of pallidal units significantly higher during singing than non-singing periods (mean ISI C.V. singing/non-singing  =  1.44, p  =  0.001, t-test). Such phasic decreases and increases of pallidal firing rate are critical for driving modulation of efferent thalamic neurons during singing [29],[30]. In contrast, the average firing rate of all LMAN units was consistently increased during singing (mean singing/non-singing  =  2.42 ± 0.42).

Typical features of Area X activity related to singing are evident in a representative recording, in which a male was presented with different female types (Fig. 1). From a relatively high and regular firing rate before singing, activity of this unit was mainly reduced during singing, with periods of phasic increases. Characteristic pauses of activity are seen in expanded periods of panel A containing final song motifs and non-singing periods (Fig. 1B, epoch indicated by color band in panel A). When aligned by a common song motif of this bird, characteristic patterns of inhibition and excitation are evident in spike timing during successive motif renditions (Fig. 1C) and in the average spike rate across all motifs (Fig. 1D, upper).

During singing directed to females, activity of AFP units consistently is less variable across renditions compared to undirected singing with no particular target [26],[27]. This distinction appears to reflect a common phasic pattern of activity modulation present in both singing types driven by input from the premotor nucleus HVC, while higher levels of activity that are less related to this pattern also occur throughout the song motif during undirected singing. Thus, across multiple renditions of a song motif, the pattern of the average firing rate is similar for both directed and undirected singing. The higher level of variability characteristic of undirected singing can occur in addition to the basic singing-related pattern in two ways. At a given location within the song motif, there can be variability in the firing rate across multiple song renditions (e.g., Fig. 1C raster plot around the end of third syllable). Such *cross-rendition variability* of the firing rate was quantified by the C.V., the standard deviation divided by the mean firing rate for each timepoint (Fig. 1D, see Materials and Methods for details). In the converse dimension, within each motif rendition the phasic instantaneous firing rate can be more or less similar to the average firing rate pattern. Such *within-rendition variability* was quantified by calculating the cross-correlation of instantaneous firing rate for each motif rendition and the average firing rate across all renditions (defined as the mean xcorr, [24]).

Across all recordings, neither of these measures of neural variability were significantly different between singing to paired compared to nonpaired females (Fig 2A, B; paired vs. nonpaired mean C.V. p  =  0.44, mean xcorr p  =  0.92). Within individual recording sessions, while the mean cross-rendition C.V. was significantly different in some experiments, though there was no clear pattern favoring one female class (C.V. significantly higher paired n  =  5/17, nonpaired n  =  2/17).

Previous behavioral experiments have suggested that model dummies of female birds are less potent at eliciting song than live females ([31],[32] but see [33] for divergent results). Thus, presentations of paired females were interspersed with presentations of dummies, to test whether such a lower potency stimulus may be reflected in higher neural variability during directed singing. For the mean cross-rendition C.V., there was no significant trend among all units for a distinction between singing directed to paired or dummy females (Fig. 2C; paired vs. dummy p  =  0.50). The mean within-rendition xcorr had some tendency to be higher during singing to paired females compared to dummies (Fig. 2D, paired vs. dummy p  =  0.02, mean ratio paired/dummy =  1.05), and was higher for paired than dummy females within all but two recordings (15/17, p  =  0.002, chi-square test).

### B. Comparison of song features typical of directed singing between different female classes

Along with reduced variability of neural activity during directed compared to undirected singing, previous work has characterized lower variability of syllable spectral structure and an increase in singing tempo during directed singing [7],[8],[11],[17],[34]. We thus compared these features, as well as several other global singing parameters that have been previously shown to be related to singing motivation, between singing directed to different female classes [7],[35]. For each singing parameter, data from single days of recording for individual birds were combined for group analyses.

Variability of spectral structure was quantified by measuring the frequency of a motif syllable component containing a sustained stable frequency (e.g., Fig. 1C, syllable element about 150 msec from onset). The cross-rendition variability of frequency was then compared between target female classes for each day. Over all experiments, there was no tendency for frequency variability to differ during singing to paired vs. nonpaired or dummy females (Fig. 3B; p  =  0.82 and 0.75).

Among global features of singing, the latency to sing, number of motifs produced, and the tempo and acceleration with which successive motifs were produced were quantified for each female presentation in which a male sang (e.g., Fig. 3A). Across all recording sessions, the strongest trend was for a higher singing tempo to paired than nonpaired or dummy females (Fig. 3C, mean ratio of tempo paired/nonpaired  =  1.12, paired/dummy  =  1.06). Although these tendencies were not significant across all recordings, (p  =  0.26 and 0.28 respectively), this reflected some variability in two components of singing rendition tempo - the duration of each song motif and the duration of inter-motif intervals (pauses). For one bird, the duration of pauses was close to significantly shorter when singing to paired than dummy females (p  =  0.014, mean pause durations paired and dummy: 552 and 780 msec). For two others, the duration of motifs sang to paired females were shorter than those sang to nonpaired females (bird A; paired/nonpaired = 476/480 msec, p  =  0.00038; bird B; paired/nonpaired  =  597/605 msec, p  =  0.0011; Mann-Whitney *U* test), and also shorter than motifs sang to dummy females for one of these (bird B; paired vs. dummy p < 0.0001, paired/dummy  =  597/608 msec).

While there *was* a clear group of recordings in which males began singing more quickly to paired females than others (Fig. 3D, note paired latencies clustered at about one second), the high variability across recordings resulted in no significant difference between female classes (p > 0.9, median ratio of latency paired/nonpaired, paired/dummy  =  0.86, 0.82 respectively). The average number of motifs per singing bout was also not significantly different between groups (not shown, paired vs. nonpaired, dummy p  =  0.36, 0.42, mean ratio motif number paired/nonpaired, paired/dummy  =  1.24, 1.12 respectively).

**Figure 4:**
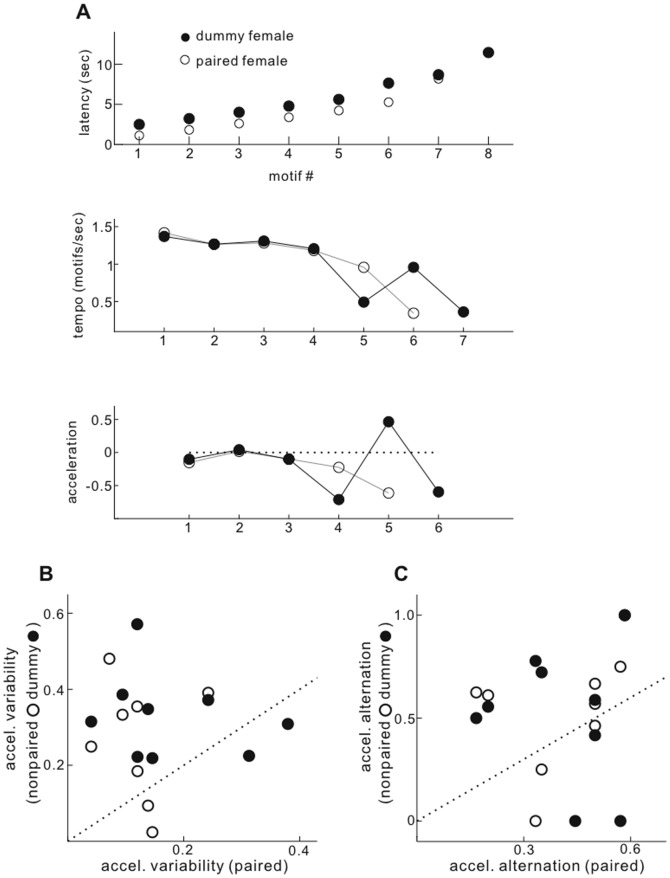
Variability of acceleration in successive song motifs is lower during singing to paired females. A, Upper, Latency of motif initiations (as in Fig. 3A) during single bouts of singing to paired and dummy females. Middle, Instantaneous tempo of motif production (1/(motif onset latency_x+1_-motif onset latency_x_). Lower, Instantaneous acceleration of motif production (tempo _x+1_-tempo_x_). **B**, Mean variability (S.D.) of motif acceleration within singing bouts. **C**, Mean level of acceleration alternation within singing bouts (fraction of successive acceleration levels that change sign: negative to positive, or reverse). For panels **B** and **C**, one point summarizes results from singing of one bird in one recording day. Quantification of acceleration features was not possible in 2 recordings for nonpaired and dummy conditions, due to the sparsity of sustained singing.

As briefly noted previously [36],[37],[38], zebra finches have some tendency to reduce their singing tempo within a song bout. We suspected that such deceleration may reflect in part a habituation of singing males to their female target, and thus could be a sensitive indicator of male courtship motivation within single bouts. In order to quantify reduction of singing tempo during successive motif renditions, we calculated the rate of motif acceleration (derivative of tempo). The average level of acceleration over all song motifs was consistently negative (mean  =  -0.13 motifs/sec^2^, p < 0.001 less than zero, t-test), with the average acceleration for paired, nonpaired and dummy females -0.13, -0.19, and -0.09 respectively. Further, the average acceleration level in the majority of singing bouts was negative (paired, nonpaired, dummy  =  0.89, 0.94, 0.82 respectively). Average acceleration levels within each recording day were not significantly different between female classes (Fig. 3E, paired vs. nonpaired, dummy p  =  0.45, 0.38, mean acceleration paired, nonpaired, dummy  =  -0.15, -0.21, -0.11 respectively). A closer examination of instantaneous acceleration levels within individual singing bouts revealed an unexpected pattern (Fig. 4A). While the simplest pattern of singing deceleration would be a monotonic slowing of successive motifs, it was more common that both decreases and increases of singing tempo occurred - thus acceleration was both negative and positive within singing bouts. For all recording sessions, about 1/3 of song motifs were produced with a faster tempo than immediately preceding ones (29 ± 3%). Further, deceleration and acceleration of singing tempo often appeared oscillatory, such that successive motifs were produced with opposite signs of acceleration (e.g., Fig. 4A lower panel, dummy female).

As two ways of quantifying the strength of such variable and oscillating accelerations, we measured the standard deviation of acceleration values within song bouts (*acceleration variability*) and the fraction of successive motifs that were alternately positive and negative, as above (*acceleration alternation*). Across the group of recording days, acceleration variability within singing bouts was nearly significantly lower for singing to paired than to nonpaired or dummy females (Fig. 4B, paired vs. nonpaired, dummy p  =  0.027, 0.029; median accel. S.D. paired/nonpaired  =  0.34, mean accel. S.D paired/dummy  =  0.61).

While a similar comparison of acceleration alternation level across recording days did not reveal clear differences between paired and other female classes (Fig. 4C; paired vs. nonpaired, dummy p  =  0.16, 0.57 sign-rank test; median ratio paired vs. nonpaired, dummy  =  0.76, 0.58), such alternating accelerations were less common when singing to paired females. While among all singing bouts, 53% of successive acceleration values crossed the zero level (alternated from acceleration to deceleration or vice versa), only 43% crossed when singing to paired females, and over half crossed when singing to other female classes (nonpaired  =  56%, dummy = 57%).

### C. Relationship of singing and neural correlates of directed singing within single recordings

**Figure 5:**
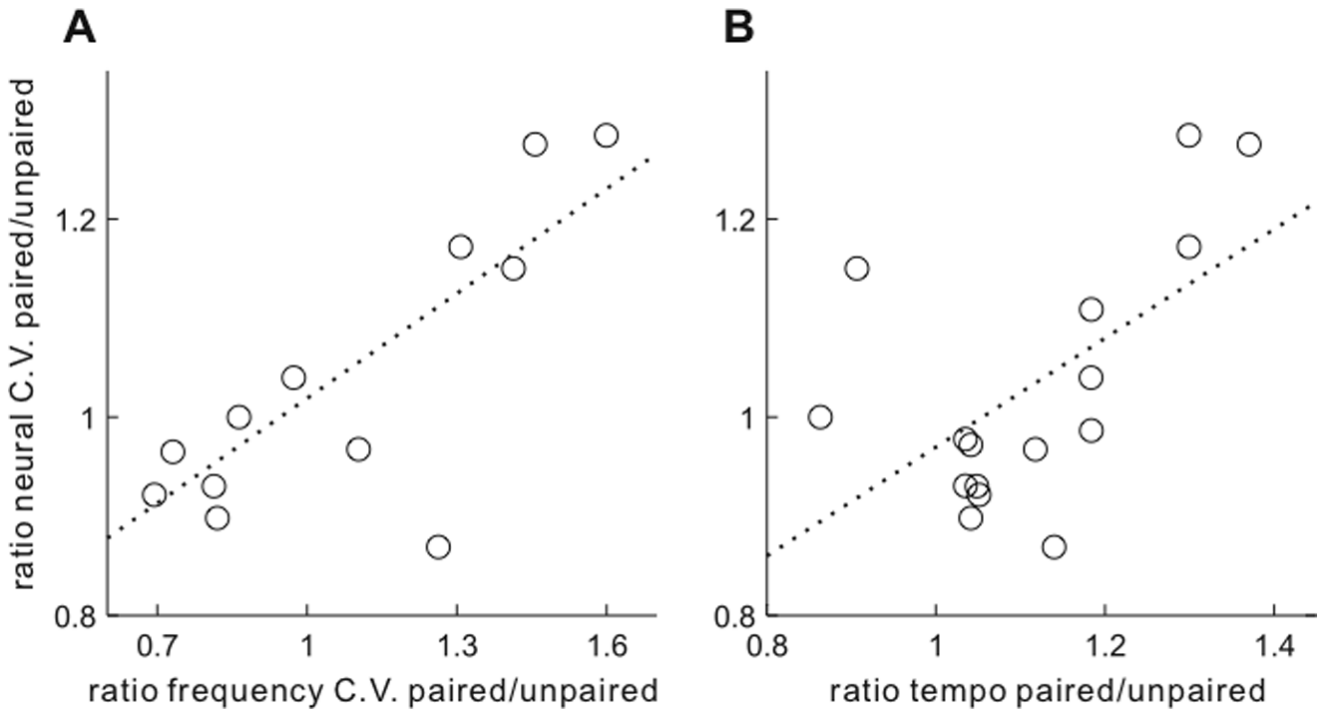
Singing features related to variability of neural activity across all recordings. A, The ratio of fundamental frequency C.V. during singing to paired relative to nonpaired females is plotted versus the ratio of mean cross-rendition C.V. of neural activity level during paired relative to nonpaired females. Values of ratio frequency C.V. greater than 1 indicate recordings in which syllable frequencies were more variable during singing to paired than nonpaired females, while values of ratio neural C.V. greater than 1 indicate recordings in which neural activity was more variable during singing to paired than nonpaired females. One recording was excluded due to lack of a constant frequency syllable element. **B**, The ratio of mean tempo during singing to paired relative to nonpaired females is plotted versus neural variability ratio as in panel **A**. For both panels, each point indicates singing and neural data from a single recording session. For sessions in which 2 units were recorded, the results from both units were combined.

We next tested whether neural correlates of directed singing were related to singing features within single recording sessions. In each recording session, the ratio of syllable frequency C.V between singing directed to paired and other female classes was used to compare song variability between them (e.g., syllable frequency C.V. for paired/nonpaired females). Similarly, in each recording session, the ratio of neural cross-rendition C.V. was used to compare neural variability during singing directed to different female classes. Among all recordings, the ratio of syllable frequency C.V. was significantly related to the ratio of cross-rendition C.V. of neural activity between paired and nonpaired singing (Fig. 5A, p  =  0.002, r  =  0.79 Pearson's correlation). During recordings in which singing was more "directed" for paired females (i.e., lower syllable frequency C.V. for paired than nonpaired), neural activity was also more "directed" (i.e., lower cross-rendition neural C.V. for paired than nonpaired). Thus, across the population of recordings, fine control of syllable frequency structure was closely linked to regularity of neural activity. The same comparison for paired vs. dummy singing conditions was similar, but not significant (p  =  0.1, r  =  0.49). A relationship between the ratio of frequency C.V. with the ratio of mean within-rendition xcorr was not clear for either paired vs. nonpaired or paired vs. dummy comparisons (p  =  0.3, 0.58).

Among global singing features, the strongest trend was a positive one between the ratio of tempo and the ratio of cross-rendition C.V. comparing paired and nonpaired singing (Fig. 5B, p  =  0.018, r  =  0.58). No other comparisons between singing features and neural variability, both cross-rendition C.V. and within-rendition xcorr, were significant.

## Discussion

The main result of this study is that within individual recordings the variability of neural activity was closely related to syllable variability, but across all recordings variability of both were not consistently dependent on female pairing status. Based on previous results that such neural variability directly controls song variability, and that low levels of both are characteristic of courtship as opposed to non-courtship singing, these results suggest that male courtship motivation may vary depending on the target female, and can be evident in singing-related AFP activity.

Within single recordings from individual males, the variability of AFP activity and song syllable structure were consistently related: when activity was less variable to one female class, syllable frequency was also less variable to that female class. Previous studies had shown that both neural and song variability are consistently lower during directed than during undirected singing [8], [12],[17],[26],[27],[39],[40],[41]. The current results extend this, by showing that within the behavior of directed singing, there can be distinct levels of neural and song structure variability. Directed songs with relatively low neural and song variability could reflect a male's high level of courtship motivation toward a target female.

The correlation of syllable and AFP neural variability dependent on female identity could be causally related. Based on a variety of approaches, the level of neural variability in the AFP appears closely related to the strength of dopaminergic input, including that from VTA [13],[14],[15],[16],[17]. Thus, the levels of VTA activity and dopamine in Area X could signal the relative preference of a male for in individual female, and subtly modulate the level of syllable frequency variability [34].

Although there was no overall trend for males to court paired or nonpaired females with higher motivational level (as defined by low neural and singing variability), among recordings such motivation appeared higher for sometimes for paired and sometimes nonpaired for females. Such labile courtship preferences may reflect individual males' personal history and current motivational state. Related to this, here and in most other studies examining physiological correlates of pair bonding, individuals were classified either as paired or nonpaired. However, it is likely that operationally paired males and females could have been relatively satisfied or unsatisfied with their randomly selected partners, which may influence their relative motivation for courtship with paired or nonpaired individuals. Female zebra finches who form a pair with a relatively preferred or nonpreferred male will reject and accept extra-pair courtship, respectively, even though they have similar number of offspring with either male type [42]. Further work to rigorously quantify pair-bond strength after extended separation will be required to address this question.

In sum, this study indicates that directed singing is not a uniform behavior, but can vary in intensity. This variation could reflect both preferences of a singing male, and the relative preference of the target female for the proposing male.

## Supporting Information

Figure S1 **Summary of Area X unit activity modulation during singing.** A, Mean unit firing rate during quiet non-singing periods (mean non-singing s.r.) is plotted versus variability of instantaneous firing rate in non-singing periods. **B**, Modulation of average firing rate during singing compared to non-singing periods. **C**, Relative variability of instantaneous spike rate during singing compared to non-singing periods. Data from one putative striatal unit (non-singing s.r.  =  9.8 Hz, singing/non-singing mean  =  4.3) is not visible at the scale used in panel B.
(TIFF)
