# Supplementary figures and images for: Singing-Related Activity in Anterior Forebrain of Male Zebra Finches Reflects Courtship Motivation for Target Females
Source: PLoS One. 2013 Nov 29;8(11):e81725. doi: 10.1371/journal.pone.0081725 (PMC3843691; doi:10.1371/journal.pone.0081725)

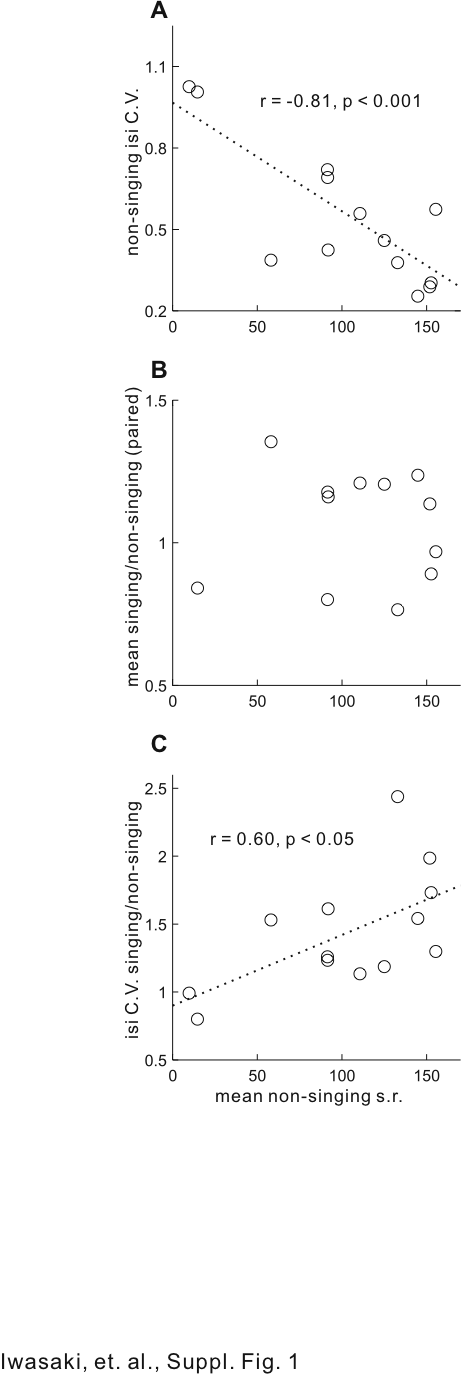

Supplement: Figure S1 — Summary of Area X unit activity modulation during singing. A, Mean unit firing rate during quiet non-singing periods (mean non-singing s.r.) is plotted versus variability of instantaneous firing rate in non-singing periods. B, Modulation of average firing rate during singing compared to non-singing periods. C, Relative variability of instantaneous spike rate during singing compared to non-singing periods. Data from one putative striatal unit (non-singing s.r. = 9.8 Hz, singing/non-singing mean = 4.3) is not visible at the scale used in panel B. (TIFF) [file pone.0081725.s001.tiff]
